# A novel smartphone augmented reality-based solution for small intracranial lesion localization with refined reference markers

**DOI:** 10.3389/fneur.2025.1566557

**Published:** 2025-07-10

**Authors:** Yu-qin Ye, Guan-yi Wang, Ying-nan Fan, Zhu-sheng Feng, Yi-bin Jia, Wei Bai, Yong-xiang Yang, Xiao-sheng He

**Affiliations:** ^1^Department of Neurosurgery, Xijing Hospital, Air Force Medical University, Xi’an, China; ^2^Department of Neurosurgery, PLA 921st Hospital, Changsha, China; ^3^Department of Emergency, Xijing Hospital, Air Force Medical University, Xi’an, China; ^4^Department of Neurosurgery, General Hospital of Western Theater Command, Chengdu, China

**Keywords:** augmented reality, smartphone, lesion localization, reference marker, neurosurgery

## Abstract

**Objectives:**

Neuronavigation is crucial for locating intracranial lesions in neurosurgery. However, it is unaffordable in numerous resource-limited areas. The emerging mobile augmented reality (AR) provides a low-cost alternative to locate lesions, but its accuracy still require improvement before widespread use. This study aimed to explore a novel smartphone AR solution for lesion localization based on a newly developed application and refined reference markers.

**Methods:**

The smartphone AR solution and standard navigation were performed to locate intracranial lesions in 38 patients. The time required for AR and navigation, the deviation between lesion center points identified by AR and navigation, and the ratio of overlap region (ROR) between the lesion locations determined by both methods, were measured, respectively, to evaluate the AR performance in preoperative planning.

**Results:**

The average time required for AR was shorter than that for navigation (256.61 ± 69.75 s vs. 454.16 ± 78.85 s, *p* < 0.05), indicating the favorable efficiency of AR. The average deviation and ROR were 3.55 ± 1.71 mm and 75.03% ± 18.56%, which were within the acceptable range of intracranial lesion surgery. The overall accurate localization rate of AR was 81.57%. Moreover, compared to the first stage of this study, the time required for AR and deviation in the second stage were significantly reduced, and ROR was notably increased (*p* < 0.05). It revealed that with the accumulation of experience, AR efficiency and accuracy were improved.

**Conclusion:**

The smartphone AR-based solution provides a practical and reliable alternative to locate small intracranial lesions, especially in settings where neuronavigation is unavailable.

## 1 Introduction

Lesion localization is the first and critical step in neurosurgery. In clinical practice, empirical line marking and neuronavigation are commonly used methods to determine the location of lesions. Since empirical marking is highly dependent on operator’s understanding of anatomy and images, the individual subjectivity may lead to potential errors and limited accuracy (1, 2). The emergence of standard neuronavigation system has greatly improved the accuracy and reliability of localization (3). Nonetheless, the high cost of navigation equipment makes them inaccessible or unaffordable in numerous developing regions. Therefore, it is urgent to explore a low-cost and practical alternative solution for intracranial lesion localization.

In recent years, visual technologies such as mixed reality, virtual reality, and augmented reality (AR) have penetrated into the field of digital medicine. AR could superimpose virtual information onto the real world to enhance the perception of objects that are difficult to see physically. It also creates immersive sensory experiences beyond reality by promoting interaction between virtual and actual environment (4–6). Accordingly, AR allows visualization of lesion within the closed cranial cavity and provides accurate location (7, 8).

The commercial head-mounted device, Microsoft’s HoloLens, have been used to assist surgical practice (9). But the information is only available to the user and cannot be shared in real time with other surgical participants who are not wearing the device. And the cost, although somewhat lower than standard neuronavigation, is still a challenge for resource-limited areas (10, 11). Recently, a few studies attempted to develop more accessible and economical AR methods for lesion localization using personal mobile devices (12, 13). However, these AR solutions depended on the registrations of blurred anatomical contours or small point-like objects, which might result in limited localization accuracy. In this study, we proposed a novel solution for locating small intracranial lesion using a newly developed smartphone AR application (app) and updated registration reference markers, and evaluated its performance by analyzing the deviation and time difference from standard navigation.

## 2 Methods

### 2.1 Patients

From September 2023 to March 2024, a total of thirty-eight patients with supratentorial lesions who accepted neurosurgical operation in the Department of Neurosurgery in Xijing Hospital were involved in this study. The age of patients ranged from 17 to 70 years old, with a mean age of 43.82 ± 15.31 years old. All lesions presented with clear boundaries in preoperative magnetic resonance (MR) or computed tomography (CT) images. All patients did not have emergency surgery indications and underwent elective surgery. The study has been approved by the Ethics Committee of Xijing Hospital, Air Force Military Medical University. Informed consent was obtained from all patients.

### 2.2 Lesion localization with smartphone AR

Initially, an experienced neurosurgeon estimated the lesion vertical projection on the scalp from surgical perspective based on the existing medical images. Three button-like markers were attached around the lesion scalp projection, serving as reference markers for AR registration. The patient then underwent thin-slice MR scan with slice thicknesses of 1.00 mm to obtain raw image data that included reference markers. Next, data was imported into the open-access image processing software 3D slicer (Version 4.11, Surgical Planning Laboratory, Harvard University, United States) to segment and reconstruct virtual 3D models of reference marker, lesion, and other structures such as veins and cerebral gyrus. Then, the models were stored in OBJ format on a platform.^1^ Afterward, the models were downloaded into the AR app Pview3D (Medinsightech Development Co., Ltd., China), which was installed on a smartphone (OnePlus 9 Pro, OnePlus Technology Co., Ltd., China) running Android 13.0. Finally, we placed the smartphone on a gimbal and started Pview3D in AR mode to activate the rear camera for registration. By coordinating the camera zoom, angle, and distance, when the virtual model of reference markers was accurately superimposed to the real markers on scalp from the assumed surgical perspective, the projection of lesion model on scalp was regarded as the lesion location determined by AR, and its boundary was outlined with a marker pen. To reduce the potential user-dependent error, AR localization in each patient was repeated by two surgeons.

### 2.3 Locating lesion with neuronavigation

The navigation system has been approved for neurosurgery and is regarded as the gold standard in frameless localization. In this study, we conducted neuronavigation in all patients to evaluate the performance of smartphone AR solution. The presurgical image data were imported into the navigation system (Stealth Station S7, Medtronic, United States) and programmed in accordance with the manufacturer’s instructions to confirm the lesion location. The navigation localization for each patient was also repeated by both surgeons.

### 2.4 Time required for AR and neuronavigation

To assess the time efficiency of AR, we recorded the time required for AR and navigation by the two surgeons, and took the mean as required-time for each method in locating each lesion. The required time refers to the duration from image data importation to completion of the lesion delineation.

### 2.5 Deviation between AR and neuronavigation

The distance between AR and navigation-determined lesion center points was measured using an electronic Vernier caliper and recorded as the deviation of AR. In addition, the lesion areas confirmed by AR and navigation, as well as the overlapping area were analyzed by ImageJ (Version 1.53a, Wayne Rasband, National Institutes of Health, United States). We calculated the ratio of overlapping area to the navigation-determined area, called the ratio of overlap region (ROR). The deviation and ROR of two surgeons were measured respectively, and the mean were calculated as the AR deviation and ROR for each lesion.

To analyze the potential influence of lesion depth on the accuracy of AR localization, the depth from the surface of brain to the shallowest boundary of lesion in each patient were measured. Spearman correlation analysis was performed to test the relationship between lesion depth and AR deviation or ROR.

Moreover, we conducted a four-fold table analysis to evaluate the diagnostic potential of AR localization, focusing on the sensitivity, specificity, accuracy and the Youden index. During the operation, we designed the incision and performed the craniotomy based on the lesion location determined by navigation. If the lesion identified by AR was entirely within the skull window, its localization was considered accurate and successful. Conversely, AR localization was considered unqualified if it extended beyond the skull window.

### 2.6 Statistical analysis

Data were presented as mean ± standard deviation (SD) and analyzed by GraphPad Prism (Version 8.3.1, GraphPad Software, San Diego, United States). The *t*-test was applied to evaluate the statistical difference between two groups. One-way analysis of variance test was used to assess the statistical difference among multiple groups. The significance level was set at 0.05.

## 3 Results

### 3.1 Characteristics of patients and intracranial lesions

Of the thirty-eight patients, twenty-two were female and sixteen were male. There were nine lesions in the frontal lobe, thirteen lesions in the parietal lobe, nine lesions in the temporal lobe, and seven lesions in the occipital lobe. The pathologies included one case each of cerebral abscess, cysticercosis, skull hemangioma, cerebral lymphoma, and astroblastoma. In addition, there were four cavernous angiomas, six gliomas, eighteen meningiomas, and five cerebral metastases. Twenty-one lesions were located in the left hemisphere, while seventeen were in the right hemisphere. The average diameter and depth of lesion were 1.89 ± 1.10 cm and 1.80 ± 1.36 cm, respectively. AR and navigation were successfully performed in all patients (Table 1).

**Table 1:** Characterization of patients and intracranial lesions.

Variable	Mean ± SD/*N*	Range/Proportion
Age (years)	43.82 ± 15.31	17–70
Gender		
Male	16	42.11%
Female	22	57.89%
Side		
Left hemisphere	21	55.26%
Right hemisphere	17	44.74%
Location		
Frontal lobe	9	23.68%
Temporal lobe	9	23.68%
Parietal lobe	13	34.22%
Occipital lobe	7	18.42%
Pathology		
Meningioma	18	47.37%
Metastasis	5	13.16%
Lymphoma	1	2.63%
Cavernous angioma	4	10.53%
Glioma	6	15.79%
Abscess	1	2.63%
Skull hemangioma	1	2.63%
Cysticercosis	1	2.63%
Astroblastoma	1	2.63%
Lesion diameter (cm)	1.89 ± 1.10	0.6–5.2
Lesion depth (cm)	1.80 ± 1.36	0–4.5
Required time of AR (s)	256.61 ± 69.75	92–533
Required time of Nav (s)	454.16 ± 78.85	183–811
Deviation between AR and Nav (mm)	3.55 ± 1.71	0–7.3
Ratio of overlapping region	75.03% ± 18.56%	36%–100%

AR, augmented reality; Nav, navigation.

### 3.2 Comparison of the time required for AR and navigation

**Figure 1:**
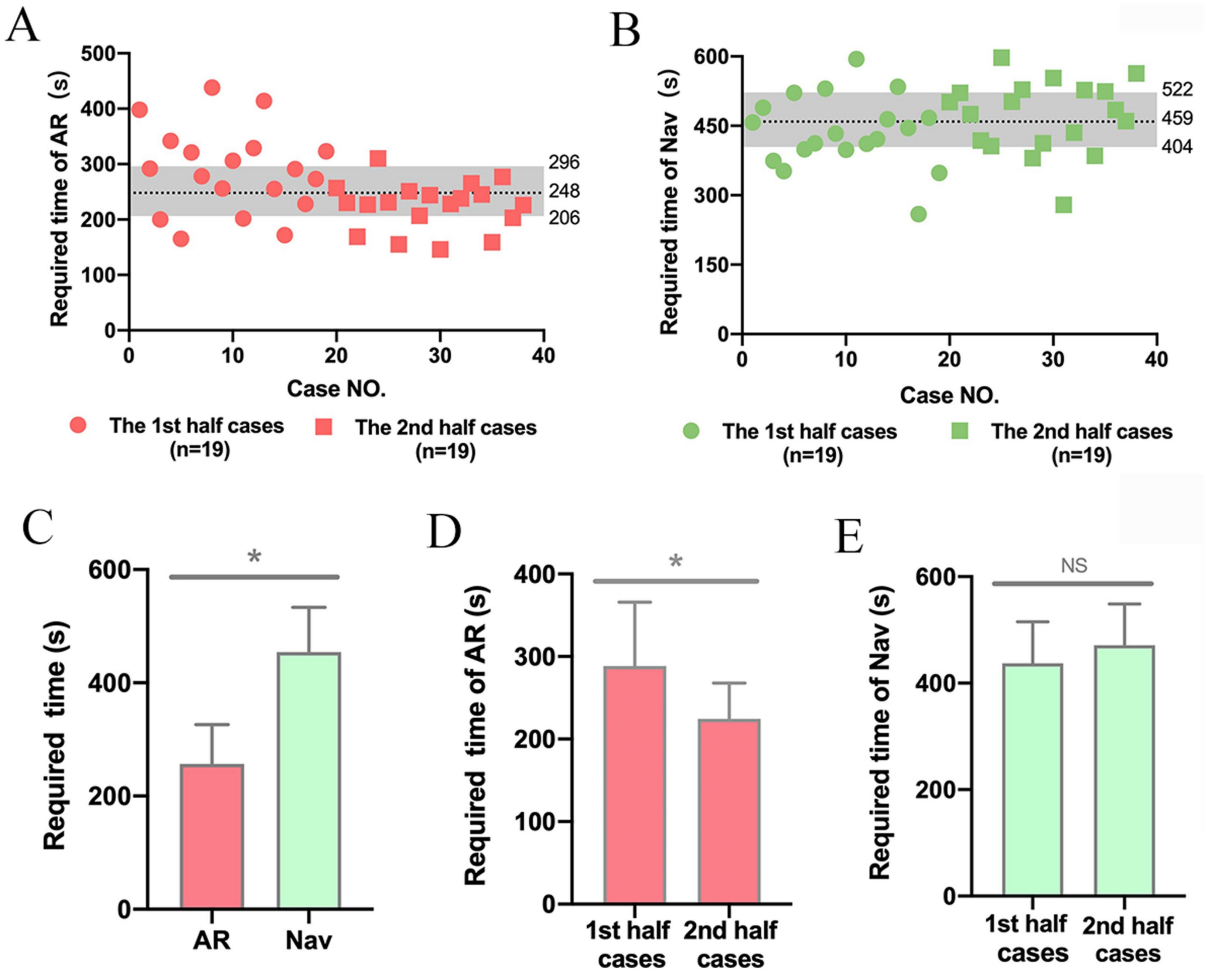
The time required for lesion localization using AR and navgation. **(A)** Scatterplot of the time required for AR in 38 cases. **(B)** Scatterplot of the time required for navigation in 38 cases. The first and third quartiles, as well as the median, were marked, respectively. **(C)** The time required for AR was significantly shorter compared to navigation. ^*^p < 0.05. **(D)** In AR localization, cases in the second half of this study took less time than those in the first half. ^*^p < 0.05. **(E)** No difference was found in the time required for navigation between the first and second stages of this study. NS, no significance, ^NS^*p* > 0.05.

The average time required for AR localization was 256.61 ± 69.75 s (Figure 1A), while navigation took an average of 454.16 ± 78.85 s (Figure 1B). There was a significant difference between the required time of AR and navigation (*p* < 0.05) (Figure 1C). Meanwhile, the time of AR localization was significantly decreased in the second 19 cases compared to the first 19 cases (224.63 ± 43.25 s vs. 288.58 ± 77.28 s, *p* < 0.05) (Figure 1D), indicating a declining trend throughout the study. However, the difference and trend were not found in the time of navigation localization (437.26 ± 78.14 s vs. 471.05 ± 77.92 s, *p* > 0.05) (Figure 1E).

### 3.3 Evaluation of locating deviation between AR and navigation

**Figure 2:**
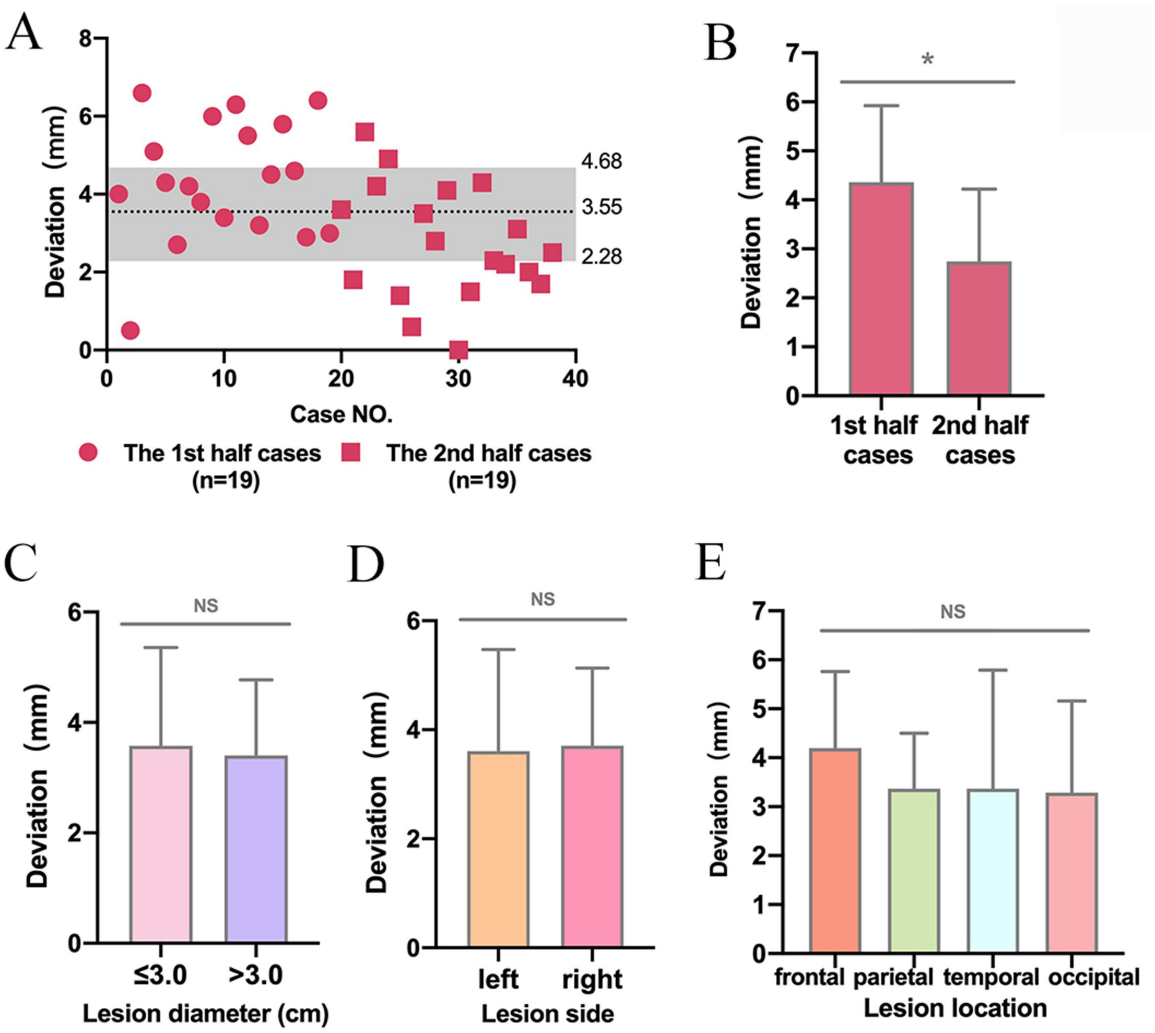
The locating deviation between AR and navigation. **(A)** Scatterplot of the deviation in 38 cases. **(B)** Deviation in the second stage of this study was less than that in the first stage. ^*^p < 0.05. **(C–E)** There was no difference in the deviation between lesions with a diameter of less than 3.0 cm and those larger than 3.0 cm, or between lesions in the left and right hemispheres, or among lesions in the frontal, parietal, temporal and occipital lobes. NS, no significance, ^NS^*p* > 0.05.

The average deviation between AR and navigation was 3.55 ± 1.71 mm (Figure 2A). In addition, the locating deviation of the second 19 cases were smaller than that of the first 19 cases (2.74 ± 1.48 mm vs. 4.36 ± 1.56 mm, *p* < 0.05) (Figure 2B). However, no significant difference was found in deviation between lesions with diameters less than 3.0 cm and those larger than 3.0 cm (3.58 ± 1.78 mm vs. 3.40 ± 1.37 mm, *p* > 0.05), or between lesions in the left and right hemispheres (3.61 ± 1.86 mm vs. 3.71 ± 1.43 mm, *p* > 0.05), or among lesions located in the frontal, parietal, temporal and occipital lobes (4.20 ± 1.56 mm vs. 3.37 ± 1.13 mm vs. 3.37 ± 2.42 mm vs. 3.29 ± 1.87 mm, *p* > 0.05) (Figures 2C–E).

**Figure 3:**
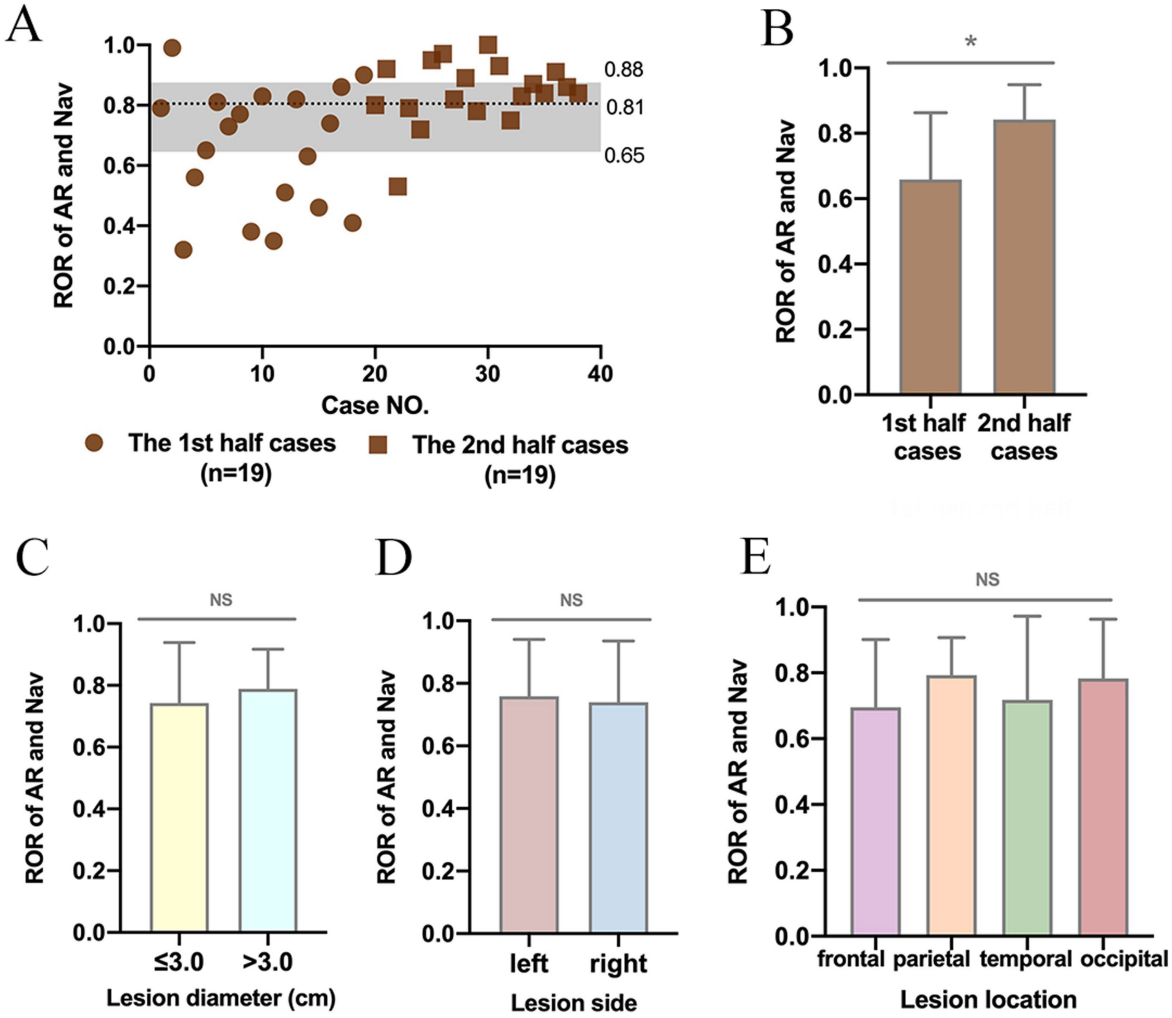
Ratio of overlap region (ROR) between lesion locations determined by AR and navigation. **(A)** Scatterplot showing the ROR in 38 cases. **(B)** ROR in the second half of this study was much higher than that in the first half. ^*^p < 0.05. **(C–E)** No significant difference was detected in ROR between lesions with diameters less than 3.0 cm and those larger than 3.0 cm, or between lesions in the left and right hemispheres, or among lesions in the frontal, parietal, and temporal occipital lobes. NS, no significance, ^NS^*p* > 0.05.

We further analyzed the ROR to evaluate the accuracy of AR localization. The average ROR was 75.03 ± 18.56% (Figure 3A). Notably, the ROR of the second 19 cases was higher than that of the first 19 cases (65.84% ± 20.41% vs. 84.21% ± 10.66%, *p* < 0.05) (Figure 3B). Nevertheless, there was no significant difference in ROR between lesions with diameters less than 3.0 cm and those larger than 3.0 cm (74.31% ± 19.52% vs. 78.83% ± 12.89%, *p* > 0.05), or between lesions in the left and right hemispheres (75.90% ± 18.15% vs. 73.94% ± 19.56%, *p* > 0.05), or among lesions in the frontal, parietal, temporal and occipital lobes (69.56% ± 20.59% vs. 79.31% ± 11.38% vs. 71.78% ± 25.46% vs. 78.29% ± 17.98%, *p* > 0.05) (Figures 3C–E).

**Figure 4:**
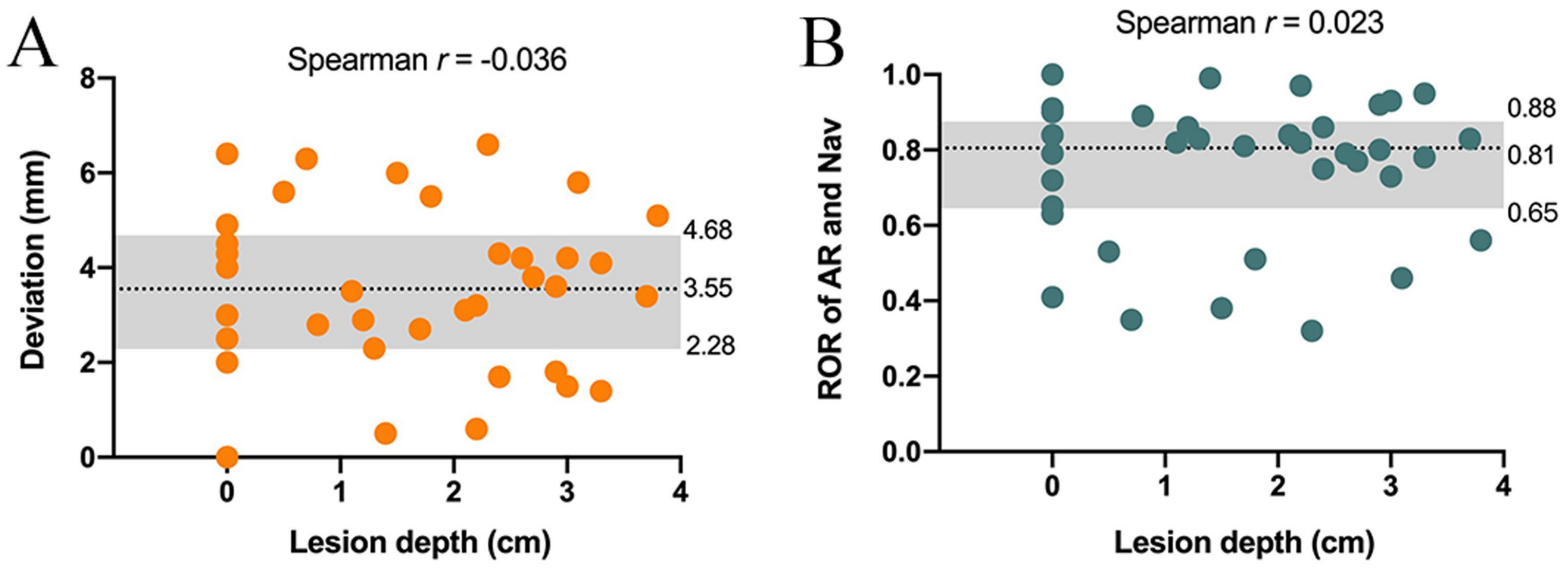
Scatterplots of locating deviation and ROR in lesions with different depths. **(A)** Spearman analysis did not find correlation between deviation and lesion depth. **(B)** There was no correlation between ROR and lesion depth. NS, no significance, ^NS^*p* > 0.05.

Furthermore, the Spearman correlation analysis showed that both the AR deviation and ROR were not correlated with lesion depth (*r* = −0.036, *p* > 0.05; *r* = 0.023, *p* > 0.05) (Figures 4A,B). Evaluation of diagnostic potential showed that 30 out of 38 lesions were successfully localized by AR solution, with a localization accuracy rate of 81.57%, sensitivity of 81.08%, and specificity of 100% (Table 2).

**Table 2:** Analysis on the accuracy rate of AR localization.

AR	Nav		Total
	Successful localization	Unsuccessful localization	
Successful localization	30	0	30
Unsuccessful localization	7	1	8
Total	37	1	38

AR, augmented reality; Nav, navigation.

### 3.4 Analysis of individual variation between two users

Comparison of the performance of both surgeons in lesion localization using AR and navigation was shown in Supplementary Figure 1 and Supplementary Table 1. There was no significant difference in required time, deviation and ROR between the two surgeons (*p* > 0.05).

### 3.5 Representative case

**Figure 5:**
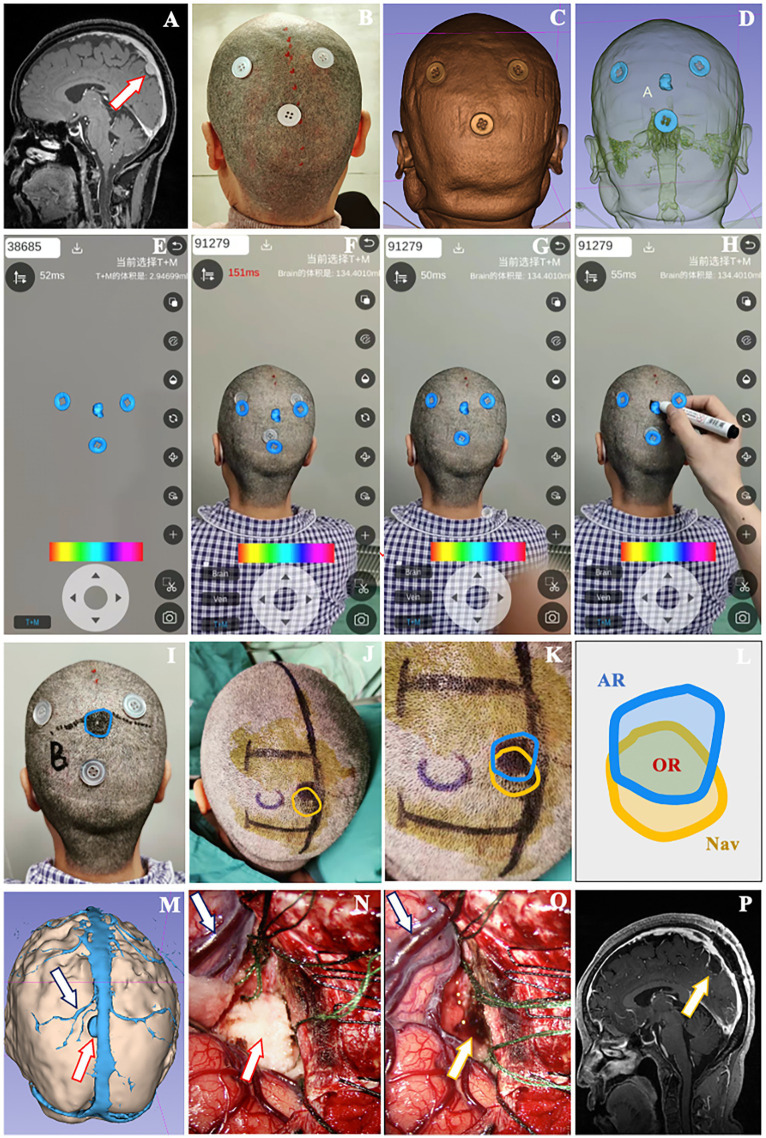
A 65-year-old female patient with a meningioma underwent localization by smartphone AR solution. **(A)** The lesion was located close to the superior sagittal sinus in the parieto-occipital area (red arrow). **(B)** Three button-like reference markers were attached around the approximate lesion scalp location before thin-slice MR scan. **(C)** Posterior view of the 3D model of reference markers and scalp. **(D)** See-through view of the cranial cavity revealing the relative relationship between markers and lesion. **(E)** The model of markers and lesion were loaded into Pview3D on a smartphone. **(F)** The model of markers was adjusted to match the real markers on scalp via coordination of the camera zoom, angle and distance in Pview3D. **(G,H)** When the virtual markers model was fully superimposed onto the actual markers, we depicted the projection of lesion model on the scalp using a marker pen and recorded it as the lesion location determined by AR. **(I)** The blue line indicated the lesion boundary identified by AR described above. **(J)** Unpon confirming the lesion boundary with neuronavigation (yellow line), a U-shaped incision was designed. **(K,L)** Schematic diagram illustrating the overlapping region (OR) located by AR and navigation. **(M)** Preoperative view of the 3D model of lesion (red arrow), neighboring veins (dark blue arrow) and sagittal sinus to simulate the surgical approach. **(N)** The lesion (red arrow), neighboring veins (dark blue arrow) and sagittal sinus were exposed during operation. **(O)** The tumor was removed (yellow arrow), with the veins (dark blue arrow) and brain tissue well protected. **(P)** Postoperative MR scan revealing the total resection of lesion (yellow arrow).

The patient is a 65-year-old woman with a meningioma in the left parieto-occipital region. External hospital images revealed that the lesion was close to the sagittal sinus and cerebral falx, with a diameter of 1.6 cm (Figure 5A). Three reference markers were attached around the approximate location of the lesion on scalp (Figure 5B). Subsequently, the patient underwent a thin-slice MR scan (Espree 1.5 T, Siemens) to obtain the raw image data with reference markers included.

Using 3D Slicer, we constructed 3D models of reference markers, lesion, brain tissue, superior sagittal sinus and veins (Figures 5C,D). After loading the models into Pview3D on a smartphone (Figure 5E), we adjusted the camera zoom, angle and distance to completely coincide the virtual marker models with the actual reference markers on scalp for registration (Figures 5F,G). Then, we depicted the projection of the lesion model on scalp with blue line and recorded it as the location identified by AR (Figures 5H,I). Following the lesion location was verified by neuronavigation and outlined with yellow line, we designed the incision in accordance with the minimally invasive principle (Figure 5J). The overlapping region (OR) between the lesion locations determined by AR and navigation was analyzed using ImageJ (Figures 5K,L).

Prior to operation, we reviewed the 3D model of lesion (red arrow), neighboring veins (dark blue arrow), sagittal sinus, and brain tissue from the scheduled surgical perspective to understand their anatomical relationship (Figure 5M). During the operation, the lesion (red arrow) was exposed to validate AR localization (Figure 5N). The neighboring veins (dark blue arrow) and brain tissue were decompressed following the resection of lesion (yellow arrow) (Figure 5O). Postoperative MR confirmed that the lesion was totally removed (yellow arrow) (Figure 5P), and the pathology report indicated a meningioma.

## 4 Discussion

As a cutting-edge visual technology, AR not only enhances the visualization of reality but also allows for extended interaction with reality. It has been employed in various fields of healthcare, including surgical assistance, anatomic training and medical education (14). Studies have shown that Microsoft’s HoloLens was used to guide the placement of external ventricular drainage for hydrocephalus with a target deviation of 4.34 mm. Compared with freehand puncture, the number of HoloLens-assisted punctures was significantly reduced (15). Other head-mounted devices, such as Epson BT-200 Glasses and MagicLeap Glasses, were applied to locate lesions in a series of simulated skull models and clinical cases. The reported deviations were 2.1 mm and 2.8 mm, respectively (16–18). However, the image and information of these wearable devices are only provided to the user and cannot be shared in real time by other participants, which may affect intraoperative communication and cooperation. The distortion of light and vision may also reduce the user’s comfort and concentration during operation. Meanwhile, the cost of the commercial AR product remains a challenge for resource-limited areas.

Recently, the personal mobile devices, such as smartphones and tablets, have attracted increasing attention from neurosurgeons who are exploring real-time shared and economical AR solution for lesion localization (19, 20). Hou et al. (21) developed an iPhone-assisted AR method to locate lesions based on the camera double exposure app FUSED and presurgical sagittal images, with an average deviation of 4.20 mm. Sina is a smartphone image-guided app that allows the overlay of transparent cross-sectional medical image on patient’s head to show the lesion location. It has been used for supratentorial tumor localization with a deviation range of 4.4 mm to 10.2 mm (22, 23). In addition, the FUSED and Sina-based AR solutions also have been used to locating the center of intracerebral hematoma for endoscopic evacuation surgery (24, 25). However, these solutions relied on a single cross-sectional 2D image to match the blurred anatomical contour and therefore could not provide stereoscopic registration and localization with high accuracy, especially for small lesions far from the midline.

In this study, based on the newly developed smartphone app Pview3D, we performed AR registration by superimposing the 3D markers model on real markers across multiple spatial dimensions to identify the lesion location. The button-like markers were made of acrylonitrile-butadiene-styrene (ABS) plastic with four inner holes in the center, which were very low-cost and easy to get. Compared to the blurred anatomical contour, the clear geometric shape, size, thickness, and inner holes of buttons facilitated precise matching between virtual and actual markers, thereby improving the accuracy of AR registration and localization. Additionally, the button markers were placed closer to lesion than the boundary of anatomical contour, making it easier for surgeons to confirm the location with less visual misunderstanding and time. Moreover, the reference markers-based registration was free of the errors caused by soft tissue displacement during anatomical contour registration.

Our primary evidence showed that the average AR deviation was 3.55 mm, which fell within the acceptable range of intracranial lesion surgery. It was superior to pervious FUSED and Sina-assisted localization that relied on 2D cross-sectional image registration, with a deviation range of 4.0 mm to 10.2 mm (21, 23). In addition, the deviation was also less than the average deviation (4.7 mm) of Unity3D-based AR solution, which performed 3D anatomical contour registration in simultaneous localization and mapping mode (12). Recently, several AR apps, such as VION, MARIN, and NeuroKeypoint, have been programed on mobile terminals to locate lesions through 3D anatomical contours or small point-like objects registration (26–28). However, the accuracy of these solutions was only evaluated on printed head models and coordinates, the actual deviation from standard navigation in presurgical planning has not been reported yet.

We employed ROR as an additional criterion to evaluate the accuracy of AR. The findings showed that among the 38 patients included in this study, 24 patients had an ROR above the average level, accounting for 63.16%. Previous study has defined the ratio of the overlapping area to the AR-determined area as the evaluation criterion, which differed from our approach of calculating ROR in this study (12). As the area determined by navigation was considered the actual location of lesion and served as the gold standard in practical operation, we defined ROR as the ratio of the overlapping area to the area located by navigation, which could provide a more intuitive reflection of AR accuracy.

In addition, compared with the first 19 cases, AR deviation in the second 19 cases was significantly reduced. Correspondingly, ROR in the second 19 cases was increased. The results suggested that with the accumulation of experience, the accuracy of AR localization has been improved. Similarly, it has been documented that after preclinical training in five simulated skull models, the proficiency and accuracy of operator in using AR can be improved (28). Additionally, there was no significant differences in deviation and ROR between the lesions of different diameters, depths, hemisphere sides, and brain lobes, indicating the stable localization performance of AR in this study. Moreover, the overall qualified localization rate of AR reached 81.57%, which suggested that the accuracy maintained at a satisfactory level.

AR took less time than navigation in the task of lesion localization, revealing that it was not a time-consuming solution, at least under the conditions of this study. Compared with other AR methods in previous studies, the time required for our AR solution was also significantly reduced (16, 28). Importantly, the localization time of the second 19 cases was much shorter than that of the first 19 cases, suggesting that this AR solution was easy to learn and implement in clinical settings. In addition, change of surgical procedure or operating room layout was not needed and no adverse events was occurred during AR localization, indicating the favorable safety of the proposed AR solution.

### 4.1 Limitation and consideration

The AR solution was implemented on a single type of smart device. Further validation across a broader range of smartphones and tablets are needed in future studies. The small sample size of this study might introduce the potential bias of selection. As a result, a larger number of cases should be recruited to achieve a more comprehensive assessment of this AR solution in future studies. In addition, the performance difference between the two surgeons showed no statistical significance in terms of the required time, deviation and ROR. However, they were from the same hospital and tested the AR solution in identical clinical environments. The findings were based on the experience from a single center, which might be subject to potential user-dependent errors. Therefore, further studies conducted by diverse teams across multiple centers would help confirm the applicability of these findings and enhance the reliability of the AR solution.

Given that smaller lesions require higher localization accuracy, lesions with large size were not chosen to test this AR solution. Due to the maximum diameter and depth of lesions in this study being 5.2 cm and 4.5 cm, the findings are restricted to supporting the effectiveness of AR in locating small and superficial lesions. Another limitation concerning this AR solution is the lack of automatic overlay feedback during AR registration. As an important metric in regression analysis, root mean square error (RMSE) reflects the average degree of difference between the measured values and actual values. The analysis of RMSE between AR and Nav localization may be a potential strategy to evaluate the accuracy and reliability of registration. Based on the Android Studio platform, we will incorporate the real-time RMSE calculation into the AR app to enable active feedback of qualified registration.

Regarding the AR intraoperative usability, we have attempted to deal with the challenge in previous surgeries. The initial experience was as follows: After the patient was positioned for surgery, a multi-directional support arm was attached on one side of the operating table. The smartphone was held at the distal end of the arm, allowing for free adjustment and AR registration. Once satisfactory localization was achieved, the smartphone position was fixed and maintained. The reference markers were then removed, and head disinfection and draping were conducted according to normal surgical procedures. Meanwhile, a transparent aseptic plastic cover was employed to wrap the smartphone and support arm to comply with surgical sterility requirements. By keeping the relative position of head and smartphone unchanged, we were able to observe the lesion location via the phone screen during surgery. However, it took a long time to find the ideal position to fix smartphone. The position not only meet the requirement of AR registration and localization, but also minimized potential interference to operator. Inspired by the electrical control principle used in neurosurgical robotic arm, we intend to retrofit the existing support arm by equipping its end with an electrically controlled fine-tuning system and laser targeting devices. With the guidance of laser, the fine-tuning system can be remotely controlled to achieve precise adjustments in X, Y, and Z directions. The optimized system is expected to simplify the positioning and registration process of smartphone AR.

## 5 Conclusion

This study provides a new, low-cost mobile AR solution capable of quickly and accurately locating small intracranial lesions. Despite some remaining challenges, the smartphone AR could simplify the localization task and assist presurgical planning with acceptable deviation, particularly for hospitals with limited resources. It also has great potential for anatomy education and surgical training among junior neurosurgeons. As AR algorithms are optimized, it will integrate more conveniently and accurately with multimodal fusion imaging, enabling operators to gain a more comprehensive understanding of lesion information. Additionally, with the development of smartphone open-source tools, the AR solution will be more efficiently integrated into the surgical workflow of operating room. This will further reduce the cost of use and enhance its global availability in clinical settings.

## Data Availability

The original contributions presented in the study are included in the article/Supplementary material, further inquiries can be directed to the corresponding authors.

## Footnotes

^1^ http://forum.persptech.net
