# Supplementary material for: A novel smartphone augmented reality-based solution for small intracranial lesion localization with refined reference markers
Source: Front Neurol. 2025 Jul 10;16:1566557. doi: 10.3389/fneur.2025.1566557 (PMC12286799; doi:10.3389/fneur.2025.1566557)
Supplement: Supplementary file 1 [file Table_1.docx]

Supplementary Material

**
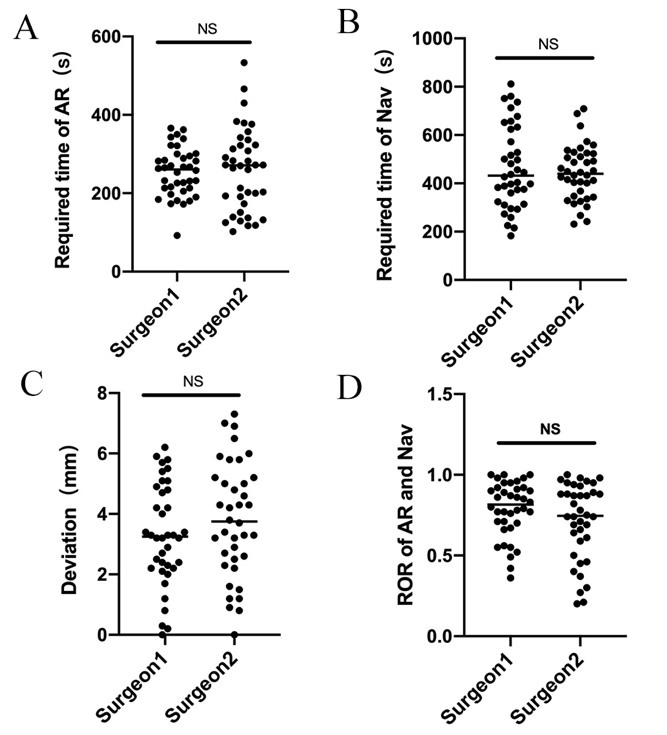
**

**Supplementary Figure 1.** Comparison of the perfromance of surgeons 1 and surgeon 2 using smartphone AR and standard navigation. (A-B) No significant difference was found in time required for AR or navigation between the two surgeons. (C-D) There was no significant difference in the deviation or ROR between the two surgeons. NS=no significance, ^NS^*P*>0.05.

**Supplementary Table 1. General demographic data of patients and localization data of intracranial lesions**

| **Case No.** | **Age(yrs) /Sex** | **Location /Side** | **Pathology** | **Diameter (cm)** | **Depth (cm)** |  | **Required time of AR (s)** | |  |  | **Required time of Nav (s)** | |  |  | **Deviation (mm)** | |  |  | **ROR** | |  |
| --- | --- | --- | --- | --- | --- | --- | --- | --- | --- | --- | --- | --- | --- | --- | --- | --- | --- | --- | --- | --- | --- |
|  |  |  |  |  |  | **Surgeon1** | | **Surgeon2** | | **Surgeon1** | | **Surgeon2** | | **Surgeon1** | | **Surgeon2** | | **Surgeon1** | | **Surgeon2** | |
| 01 | 26F | PL/R | Meningioma | 2.2 | 0.0 | 366 | | 430 | | 389 | | 525 | | 4.8 | | 3.2 | | 71% | | 87% | |
| 02 | 55F | OL/L | Meningioma | 1.1 | 1.4 | 205 | | 379 | | 572 | | 406 | | 0.2 | | 0.8 | | 100% | | 98% | |
| 03 | 64M | FL/R | Metastasis | 2.4 | 2.3 | 227 | | 173 | | 481 | | 267 | | 5.9 | | 7.3 | | 42% | | 20% | |
| 04 | 34F | FL/R | Meningioma | 4.1 | 3.8 | 301 | | 383 | | 375 | | 329 | | 4.2 | | 6.0 | | 67% | | 45% | |
| 05 | 18F | TL/L | Meningioma | 1.6 | 0.0 | 213 | | 117 | | 404 | | 638 | | 4.0 | | 4.6 | | 71% | | 59% | |
| 06 | 52F | FL/R | Meningioma | 1.2 | 1.7 | 300 | | 342 | | 225 | | 573 | | 2.1 | | 3.3 | | 87% | | 75% | |
| 07 | 47F | Tl/L | Lymphoma | 1.5 | 3.0 | 265 | | 291 | | 456 | | 368 | | 3.2 | | 5.2 | | 77% | | 69% | |
| 08 | 46M | PL/R | CA | 0.9 | 2.7 | 343 | | 533 | | 713 | | 347 | | 3.4 | | 4.2 | | 85% | | 69% | |
| 09 | 29M | TL/L | Glioma | 1.9 | 1.5 | 321 | | 191 | | 360 | | 506 | | 6.2 | | 5.8 | | 36% | | 40% | |
| 10 | 61M | OL/L | Glioma | 5.2 | 3.7 | 289 | | 323 | | 310 | | 486 | | 2.5 | | 4.3 | | 92% | | 74% | |
| 11 | 22M | TL/R | Glioma | 2.2 | 0.7 | 267 | | 137 | | 736 | | 452 | | 5.7 | | 6.9 | | 49% | | 21% | |
| 12 | 70M | PL/R | Metastasis | 1.2 | 1.8 | 350 | | 308 | | 293 | | 529 | | 5.1 | | 5.9 | | 56% | | 46% | |
| 13 | 61F | OL/L | CA | 1.0 | 2.2 | 362 | | 466 | | 397 | | 445 | | 3.3 | | 3.4 | | 98% | | 66% | |
| 14 | 39F | PL/R | Meningioma | 2.4 | 0.0 | 197 | | 313 | | 516 | | 412 | | 4.2 | | 4.8 | | 76% | | 50% | |
| 15 | 35M | FL/L | Abscess | 2.0 | 3.1 | 226 | | 118 | | 624 | | 426 | | 5.1 | | 6.5 | | 55% | | 37% | |
| 16 | 40F | FL/L | Meningioma | 3.4 | 4.5 | 322 | | 260 | | 398 | | 492 | | 4.9 | | 4.3 | | 66% | | 82% | |
| 17 | 54F | PL/R | Meningioma | 1.6 | 1.2 | 253 | | 203 | | 183 | | 335 | | 3.3 | | 2.5 | | 77% | | 95% | |
| 18 | 50M | OL/L | Meningioma | 1.0 | 0.0 | 282 | | 264 | | 500 | | 434 | | 5.8 | | 7.0 | | 52% | | 30% | |
| 19 | 31F | FL/R | Meningioma | 1.4 | 0.0 | 270 | | 376 | | 295 | | 401 | | 2.7 | | 3.3 | | 83% | | 97% | |
| 20 | 54F | PL/L | Meningioma | 4.7 | 2.9 | 231 | | 283 | | 677 | | 325 | | 2.2 | | 5.0 | | 89% | | 71% | |
| 21 | 25F | TL/R | CA | 0.6 | 2.9 | 258 | | 202 | | 811 | | 231 | | 2.4 | | 1.2 | | 86% | | 98% | |
| 22 | 47M | TL/L | Glioma | 1.1 | 0.5 | 213 | | 125 | | 427 | | 523 | | 5.4 | | 5.8 | | 79% | | 27% | |
| 23 | 67F | PL/L | Metastasis | 1.4 | 2.6 | 172 | | 282 | | 374 | | 462 | | 4.7 | | 3.7 | | 70% | | 88% | |
| 24 | 21F | FL/L | SH | 1.2 | 0.0 | 284 | | 336 | | 497 | | 315 | | 5.5 | | 4.3 | | 55% | | 89% | |
| 25 | 58M | PL/L | Glioma | 1.3 | 3.3 | 190 | | 272 | | 760 | | 434 | | 1.2 | | 1.6 | | 96% | | 94% | |
| 26 | 65F | TL/L | Metastasis | 1.1 | 2.2 | 181 | | 129 | | 445 | | 559 | | 0.3 | | 0.9 | | 100% | | 93% | |
| 27 | 53F | OL/L | Meningioma | 1.0 | 1.1 | 232 | | 270 | | 652 | | 404 | | 3.2 | | 3.8 | | 90% | | 74% | |
| 28 | 36F | PL/R | Meningioma | 2.1 | 0.8 | 282 | | 132 | | 273 | | 487 | | 2.4 | | 3.2 | | 91% | | 87% | |
| 29 | 42M | PL/L | Glioma | 2.4 | 3.3 | 295 | | 193 | | 314 | | 510 | | 3.2 | | 5.0 | | 95% | | 61% | |
| 30 | 23M | TL/L | Meningioma | 1.8 | 0.0 | 92 | | 200 | | 657 | | 449 | | 0 | | 0 | | 100% | | 100% | |
| 31 | 63M | TL/R | CA | 3.9 | 3.0 | 184 | | 272 | | 215 | | 343 | | 0.8 | | 2.2 | | 98% | | 88% | |
| 32 | 62F | OL/R | Metastasis | 1.0 | 2.4 | 263 | | 213 | | 324 | | 546 | | 3.4 | | 5.2 | | 86% | | 64% | |
| 33 | 45M | PL/R | Cysticercosis | 1.1 | 1.3 | 173 | | 357 | | 751 | | 303 | | 2.0 | | 2.6 | | 92% | | 74% | |
| 34 | 28F | PL/L | Meningioma | 3.2 | 4.1 | 339 | | 151 | | 528 | | 242 | | 2.9 | | 1.5 | | 78% | | 96% | |
| 35 | 33M | FL/L | Meningioma | 1.6 | 2.1 | 216 | | 102 | | 633 | | 415 | | 3.3 | | 2.9 | | 80% | | 88% | |
| 36 | 42F | FL/R | Meningioma | 1.0 | 0.0 | 232 | | 322 | | 259 | | 709 | | 1.7 | | 2.3 | | 95% | | 87% | |
| 37 | 17M | OL/L | Astroblastoma | 1.7 | 2.4 | 267 | | 139 | | 383 | | 537 | | 2.2 | | 1.2 | | 77% | | 95% | |
| 38 | 50F | PL/R | Meningioma | 1.5 | 0.0 | 180 | | 272 | | 437 | | 689 | | 2.3 | | 2.7 | | 90% | | 78% | |

*F: female, M: male, PL: parietal lobe, OL: occipital lobe, FL: frontal lobe, TL: temporal lobe, R: right, L: left, CA: cavernous angioma, SH: skull hemangioma, AR: augmented reality method, Nav: navigation system, ROR: ratio of overlapping region.*
